# Metabolic Radiomics for Pretreatment ^18^F-FDG PET/CT to Characterize Locally Advanced Breast Cancer: Histopathologic Characteristics, Response to Neoadjuvant Chemotherapy, and Prognosis

**DOI:** 10.1038/s41598-017-01524-7

**Published:** 2017-05-08

**Authors:** Seunggyun Ha, Sohyun Park, Ji-In Bang, Eun-Kyu Kim, Ho-Young Lee

**Affiliations:** 1 Department of Nuclear Medicine, Seoul National University College of Medicine, Seoul, Korea; 2 Department of Molecular Medicine and Biopharmaceutical Sciences, Graduate School of Convergence Science and Technology, and College of Medicine or College of Pharmacy, Seoul National University, Seoul, Korea; 3 Department of Nuclear Medicine, Seoul National University Bundang Hospital, Gyeonggi-do, Korea; 4 Department of General Surgery, Seoul National University Bundang Hospital, Gyeonggi-do, Korea; 5 Cancer Research Institute, Seoul National University, Seoul, Korea

## Abstract

Radiomics has been spotlighted as imaging biomarker for estimation of intratumoral heterogeneity (ITH) which is regarded as the main reason for resistance to tumor treatment. Although a number of studies has shown clinical evidences that separate measurement of metabolic ITH by texture features (TFs) on ^18^F-fluorodeoxyglucose positron emission tomography/computed tomography (^18^F-FDG PET/CT) has prognostic ability in various tumors, there has been no consensus regarding the best parameter representing ITH. Besides, it is yet uncertain that TFs are useful for estimation of histopathologic markers, prediction of response to neoadjuvant chemotherapy (NAC), or prognostic ability in breast cancer. To depart from the traditional approach, we evaluated the clinical usefulness of integrated metabolic radiomics using unsupervised clustering with 109 TFs measured from pretreatment ^18^F-FDG PET/CT scans of 73 patients with locally advanced breast cancer (LABC) underwent NAC before surgery. Our study shows that metabolic radiomics patterns of LABC are associated with Ki67 expression, achievement of pathologic complete response after NAC, and risk of recurrence. Integrated metabolic radiomics has potential for clinically relevant pretreatment biomarker with predictive and prognostic ability for personalized management in LABC.

## Introduction

Neoadjuvant chemotherapy (NAC) allows complete surgical resection by downstaging tumors and is now a standard treatment strategy in patients with locally advanced breast cancer (LABC). A pathologic complete response (pCR) after NAC indicates a better prognosis for patients with LABC^1^. There is much evidence that uptake on ^18^F-fluorodeoxyglucose (FDG) positron emission tomography (PET)/computed tomography (CT) scans correlates with histopathologic markers, response to treatment, and prognosis in breast cancer^2–4^. The usefulness of decrease in PET parameters between pretreatment and interim ^18^F-FDG PET/CT in early prediction of a pCR has been reported in LABC treated by NAC^5, 6^. However, pCR can only be predicted after the start of NAC and additional radiation exposure is involved.

Texture analysis, developed for image pattern recognition, has been identified as a tool for “radiomics” on medical images in recent years^7^. Radiomics contains all mineable data from medical images for clinical decision support. There have been reports that certain texture features (TFs) indicating metabolic intratumoral heterogeneity (ITH) are good prognostic markers of the likely response of the tumor to treatment and of patient survival^8–10^. Recently, success has been achieved in decoding tumor phenotypes by combining hundreds of features on CT images^11, 12^.

We hypothesized that tumors with distinctive metabolic radiomics patterns may have certain clinical characteristics. To test this theory, unsupervised clustering on pretreatment ^18^F-FDG PET/CT scans was applied to LABC tumors as part of an integrated approach to metabolic radiomics. We then investigated the relationship between tumor clusters (TCs), histopathologic characteristics, tumor response to NAC, and risk of recurrence.

## Results

### Patient Demographics

**Figure 1:**
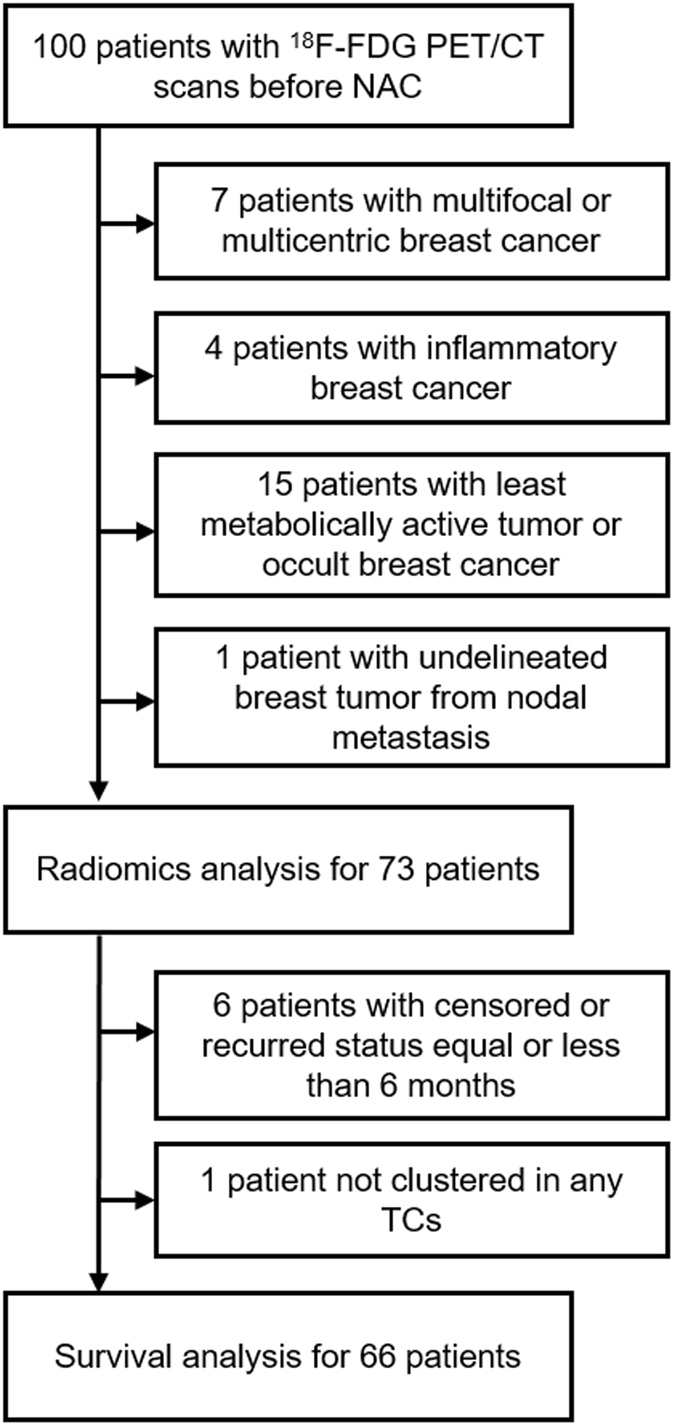
Inclusion and exclusion criteria for the study population. Seventy-three patients were involved in radiomics analysis. Sixty-six patients were involved in survival analysis. Abbreviations: NAC, neoadjuvant chemotherapy; TCs, tumor clusters.

Seventy-three patients with LABC who satisfied inclusion and exclusion criteria were included in this retrospective study (Fig. 1). The patient demographic characteristics are summarized in Table 1. The median patient age was 48 (24–76) years. Most cases (94.5%) were invasive ductal carcinoma. The clinical stage was II in 38 cases and III in 35 cases. The clinical subgroups were hormone receptor (HR)-positive/human epidermal growth factor receptor 2 (HER2)-negative in 25 cases, HER2-positive in 18 cases, and triple-negative in 25 cases. Five cases with moderate HER2 staining were unclassified due to missed fluorescence *in situ* hybridization results. Seventeen cases (23.2%) achieved a pCR. Recurrences were observed in 4 cases (6.1%), and median DFS was 25 (16–57) months.

**Table 1:** Patient Demographic Characteristics.

	Numbers
Patients (n)	73
Median age (range), years	48 (24–76)
Tumor histology	69 IDC, 1 ILC, 2 MpC, 1 MC
Clinical T stage*	
T1	0
T2	44 (60.3%)
T3	24 (32.9%)
T4	5 (7.8%)
Clinical N stage*	
N0	20 (27.4%)
N1	30 (41.1%)
N2	17 (23.3%)
N3	6 (8.2%)
Stage*	
IIa	13 (17.8%)
IIb	25 (34.2%)
IIIa	24 (32.9%)
IIIb	4 (5.5%)
IIIc	6 (8.2%)
Clinical subtype^†^	
HR-positive/HER2-negative	25 (36.8%)
HER2-positive	18 (26.5%)
TNBC	25 (36.8%)
Surgery	
BCS	36 (49.3%)
Mastectomy	37 (50.7%)
Response to NAC	
pCRs	17 (23.3%)
Non-pCRs	56 (76.7%)
Recurrence^†^	4 (5.5%)
DFS (range), months^‡^	25 (16–57)

*Staging was defined according to the American Joint Committee on Cancer system 7^th^ edition; ^†^Five cases with moderate HER2 staining were excluded from subgroup analysis due to lack of HER2 FISH result; ^‡^Recurrence and DFS was about 66 cases included in survival analysis. Notes: IDC, invasive ductal carcinoma; ILC, invasive lobular carcinoma; MpC, metaplastic carcinoma; MC, mucinous carcinoma; HR, hormone receptor; HER2, human epidermal growth factor receptor 2; TNBC, triple-negative breast cancer; BCS, breast conserving surgery; NAC, neoadjuvant chemotherapy; pCR, pathological complete response; DFS, disease-free-survival.

### Correlations between Texture Features

**Figure 2:**
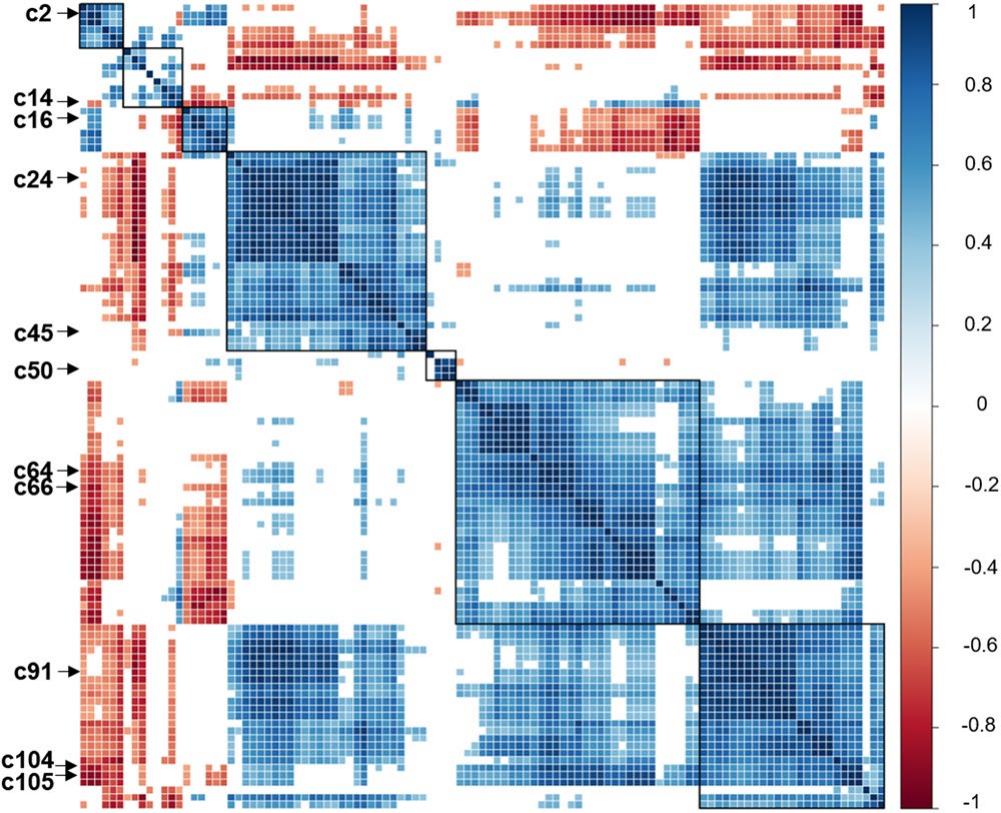
Correlogram after multiple comparison correction (*P* < 0.0005). Correlogram shows close association of TFs to each other. Correlation coefficients are expressed by color scale from red to blue. Representative TFs are marked on the correlogram. Notes: c2, NL_Homogeneity^GLCM^; c14, ZP^GLSZM^; c16, Entropy^TFCCM^; c24, MTV; c45, HILZE^GLSZM^; c50, Skewness; c64, SUV_max_; c66, NL_Dissimilarity^GLCM^; c91, TLG; c104, CV; c105, NL_Entropy^GLCM^. Abbreviations: TFs, texture features; NL, normalized; GLCM, gray level co-occurrence matrix; ZP, zone percentage; GLSZM, gray level size zone matrix; TFCCM, texture feature coding co-occurrence matrix; HILZE, high-intensity large-zone emphasis.

Correlations between TFs were expressed in an intuitive way, with the correlogram considering multiple comparison correction (Fig. 2) or not (Supplementary Fig. S1). Maximum of standardized uptake value (SUV_max_) and metabolic tumor volume (MTV) were correlated with 68 TFs (62.3%) and 45 TFs (41.3%), respectively. Only 17 TFs (15.6%) were correlated with neither SUV_max_ nor MTV. When disregarding multiple comparison correction (*P* < 0.05), only five TFs (4.6%) were correlated with neither SUV_max_ nor MTV.

### Unsupervised Tumor Clustering using Radiomics Pattern

**Figure 3:**
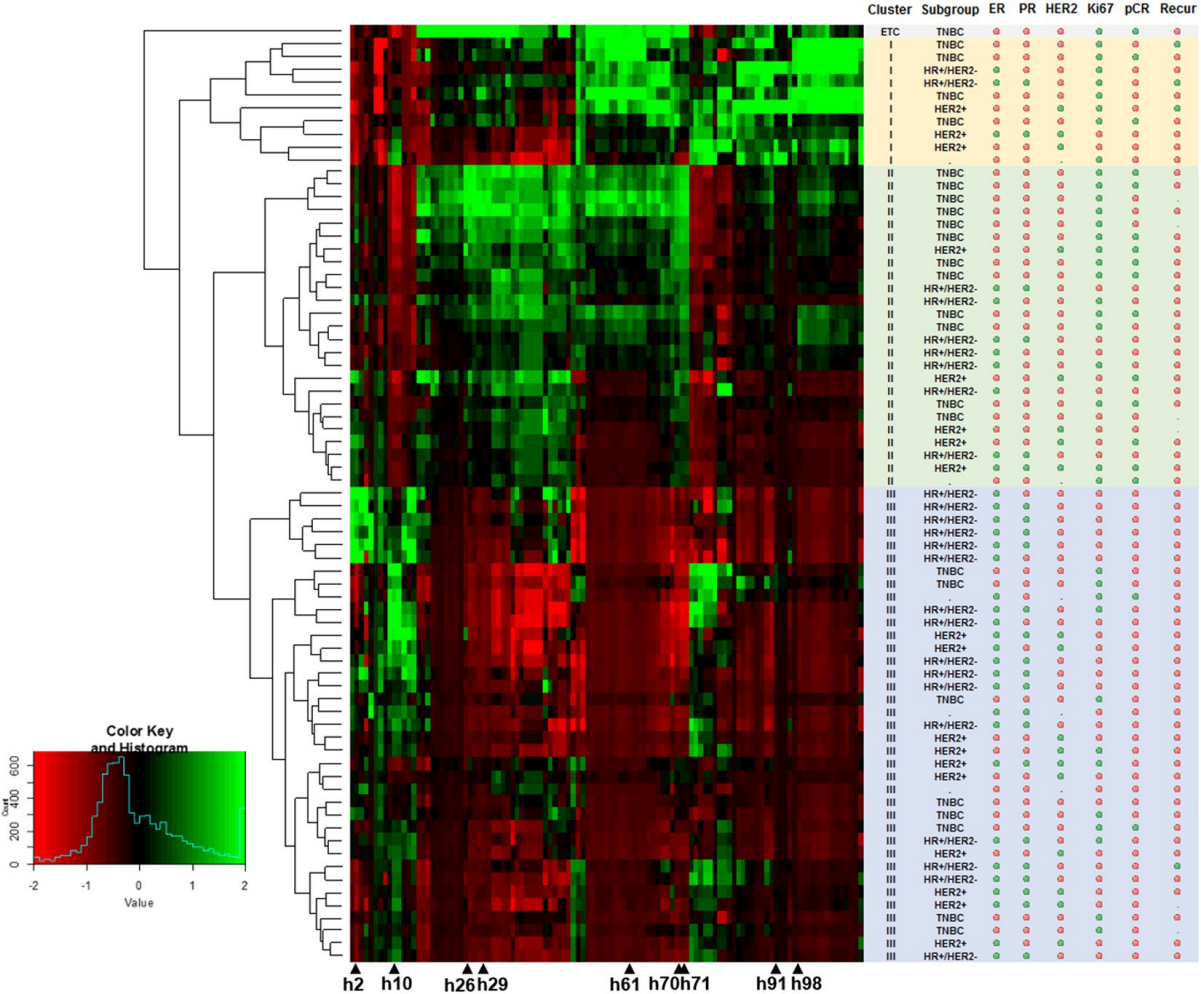
Unsupervised radiomics heat map with 109 texture features. Three individual tumor clusters (TCs) with distinctive metabolic radiomics patterns were identified after unsupervised clustering. Notes: Row, cases; column, texture features or clinical information; green circle, positive or high expression; red circle, negative or low expression; h2, ZP^GLSZM^; h10, NL_Homogeneity^GLCM^; h26, NL_Dissimilarity^GLCM^; h29, SUV_max_; h61, TLG; h70, CV; h71, NL_Entropy^GLCM^; h91, HILZE^GLSZM^; h98, MTV. Abbreviations: TNBC, triple negative breast cancer; HR, hormone receptor; ER, estrogen receptor; PgR, progesterone receptor; human epidermal growth receptor 2, HER2; ZP, zone percentage; GLSZM, gray level size zone matrix; NL, normalized; GLCM, gray level co-occurrence matrix; SUV_max_, maximum of standardized uptake value; HILZE, high-intensity large-zone emphasis; MTV, metabolic tumor volume.

We assessed TCs by metabolic radiomics patterns via unsupervised clustering. Unsupervised clustering resulted in 3 TCs (Fig. 3) and a separate case not included in any TCs (Supplementary Fig. S2). There were 10, 25, and 37 cases of TC I, II, and III, respectively. We assessed the metabolic characteristics of the unsupervised TCs by several representative TFs as follows: SUV_max_, MTV, total lesion glycolysis (TLG), coefficient of variation (CV), normalized entropy measured from gray level co-occurrence matrix (NL_Entropy^GLCM^), normalized homogeneity from gray level co-occurrence matrix (NL_Homogeneity^GLCM^), zone percentage from gray level size zone matrix (ZP^GLSZM^), and skewness (Table 2). The TFs were significantly different between the 3 TCs (all *P* ≤ 0.001). TC 1 had a large tumor size (MTV; 62.9 [25.2–158.8]), high SUV_max_ (13.2 [5.0–22.1]), and high ITH as high CV (0.42 [0.19–0.66]) and high NL_Entropy^GLCM^ (5.4 [3.4–5.9]). Like TC I, although TC II had a medium tumor size (MTV; 18.4 [4.2–43.3]), it had a high SUV_max_ (13.3 [9.2–24.6]), and high ITH (CV; 0.45 [0.35–0.60], and NL_Entropy^GLCM^; 5.8 [5.1–7.0]). TC III had a small tumor size (MTV; 2.9 [0.8–11.9]), low SUV_max_ (5.8 [3.7–9.7]), and low ITH (CV; 0.21 [0.10–0.38]), and NL_Entropy^GLCM^ (3.7 [2.5–5.0]). Representative cases of TC I-III groups can be found as Supplementary Fig. S3.

**Table 2:** Characteristics of the Tumor Clusters.

	Tumor cluster I^†^	Tumor cluster II^†^	Tumor cluster III^†^	*P*-value
Total^†^	10	25	37	—
SUV_max_	13.2 (5.0–22.1)	13.3 (9.2–24.6)	5.8 (3.7–9.7)	<0.001**
SUV_mean_	4.9 (3.1–7.2)	6.1 (5.0–10.4)	3.6 (2.9–4.6)	<0.001**
MTV	62.9 (25.2–158.8)	18.4 (4.2–43.3)	2.9 (0.8–11.9)	<0.001**
TLG	318.1 (93.9–945.5)	120.1 (21.5–449.1)	12.1 (3.1–43.1)	<0.001**
CV	0.42 (0.19–0.66)	0.45 (0.35–0.60)	0.21 (0.10–0.38)	<0.001**
NL_Entropy^GLCM^	5.4 (3.4–5.9)	5.8 (5.1–7.0)	3.7 (2.5–5.0)	<0.001**
NL_Homogeneity^GLCM^	0.37 (0.30–0.52)	0.28 (0.21–0.33)	0.43 (0.28–0.60)	<0.001**
ZP^GLSZM^	0.09 (0.02–0.18)	0.31 (0.17–0.43)	0.20 (0.04–0.67)	<0.001**
Skewness	1.1 (−0.1–2.2)	0.54 (0.0–1.6)	0.7 (−0.1–1.2)	<0.001*

*Statistically significant: *P*-values less than 0.05; **statistically significant after Bonferroni’s correction (*P* < 0.0005); ^†^tumor clusters were classified by unsupervised clustering using radiomics patterns; ^‡^one case not classified to any clusters was excluded in this analysis. Notes: SUV_max_, maximum of standardized uptake value; SUV_mean_, mean of SUV; MTV, metabolic tumor volume; TLG, total lesion glycolysis; CV, coefficient variance; NL, normalized; GLCM, gray level co-occurrence matrix; ZP, zone percentage.

### Histopathologic Characteristics of the Tumor Clusters

To characterize the unsupervised TCs, we investigated the expression of histopathologic factors, i.e., estrogen receptor (ER), progesterone receptor (PgR), HER2, and Ki67 (Table 3). The only significant difference after multiple comparison correction was Ki67 expression (*P* = 0.006). The ER and PgR were expressed at relatively higher levels in TC III than in TC I and TC II, but the difference was only statistically significant before multiple comparison correction (all *P* = 0.018). There was no statistically significant difference in HER2 expression between the unsupervised TCs (*P* = 0.688).

**Table 3:** Relationship between the Tumor Clusters and Histopathological Characteristics.

	Tumor cluster I	Tumor cluster II	Tumor cluster III	*P*-value
Total^†^	10	25	37	—
ER-positive	3 (30%)	8 (32%)	24 (65%)	0.018*
PgR-positive	2 (20%)	4 (16%)	18 (49%)	0.018*
HER2-positive^‡^	3 (33%)	5 (21%)	10 (29%)	0.688
High Ki67 (≥30%)	8 (80%)	16 (64%)	12 (32%)	0.006**

*Statistically significant: *P*-values less than 0.05; **statistically significant after Bonferroni’s correction (*P* < 0.013); ^†^one case not classified to any clusters was excluded in this analysis; ^‡^five cases with moderate HER2 staining were excluded from subgroup analysis due to lack of HER2 FISH results.

Notes: ER, estrogen receptor; PgR, progesterone receptor; HER2, human epidermal growth factor receptor 2.

### Predictors of a Pathologic Complete Response

The pCR rates were significantly different between the TC I, II, and III groups (20.0%, 48.0%, and 5.4%, respectively; *P* < 0.001). Univariate analysis revealed that the TC II (odds ratio [OR] 9.923, *P* < 0.001), ER-negative (OR 10.764, *P* < 0.001), PgR-negative (OR 11.148, *P* = 0.003), and high Ki67 (OR 11.587, *P* < 0.001) groups were significantly associated with achievement of a pCR. The TC II (OR 12.984, *P* = 0.003) and ER-negative (OR 12.607, *P* = 0.046) groups were still significant in multivariate analysis. High Ki67 expression (OR 11.051, *P* = 0.051) had intermediate significance. Table 4 summarizes the results of univariate and multivariate analysis.

**Table 4:** Logistic Regression Analysis for Pathological Complete Remission.

Parameters	Univariate analysis			Multivariate analysis		
	OR	95% CI	*P*-values	OR	95% CI	*P*-values
Age (≥50)	0.752	0.251–2.256	0.611	—	—	—
Clinical Stage II *v*. III	1.969	0.640–6.062	0.238	—	—	—
Lower T stage (cT2)	0.783	0.253–2.418	0.670	—	—	—
LN metastasis-N0^§^	1.139	0.343–3.778	0.832	1.764	0.277–11.236	0.548
ER-negative^§^	10.764	2.242–51.546	<0.003*	12.607	1.045–152.070	0.046*
PgR-negative	11.148	1.380–90.090	0.024*	—	—	—
HER2-positive^†,§^	1.010	0.277–3.706	0.985	5.311	0.560–50.328	0.146
High Ki67 (≥30%)^§^	11.587	2.410–55.556	<0.002*	11.051	0.985–123.967	0.051
Tumor clusters^‡^ (compared with TC III)						
-TC I	4.375	0.533–35.912	0.169	—	—	—
-TC II	16.154	3.175–82.177	<0.001*	—	—	—
TC II vs. others^§^	9.923	2.730–36.066	<0.001*	12.984	2.370–71.134	0.003*

*Statistically significant: *P*-values less than 0.05; ^†^five cases with moderate HER2 staining were excluded from subgroup analysis due to lack of HER2 FISH results; ^‡^one case not classified to any clusters was excluded in this analysis; ^§^parameters included in the multivariate analysis. Notes: OR, odds ratio; CI, confidence interval; TC, tumor cluster; vs., versus; ER, estrogen receptor; PgR, progesterone receptor; HER2, human epidermal growth factor receptor 2.

### Recurrence Risk for the Tumor Clusters

Three of the 4 recurrences (30.0%) were in the TC I group (n = 10); the other one (2.9%) was in the TC III group (n = 35). No recurrences occurred in the TC II group (n = 21) during follow-up. All recurrences were non-pCRs. Mean DFS was 35.3 months (95% confidence interval [CI] 29.5–41.1) in the TC I group, 55.0 months (95% CI 55.0–55.0) in the TC II group, and 39.2 months (95% CI 37.7–40.7) in the TC III group. There was a statistically significant difference in DFS between the unsupervised TC I, II, and III groups (*P* = 0.001, Supplementary Fig. S4a). When we compared DFS of binary groups as the TC I to the others (mean DFS 54.6 months [95% CI 50.0–59.1]), the TC I group had a worse prognosis (*P* < 0.001, Supplementary Fig. S4b).

**Figure 4:**
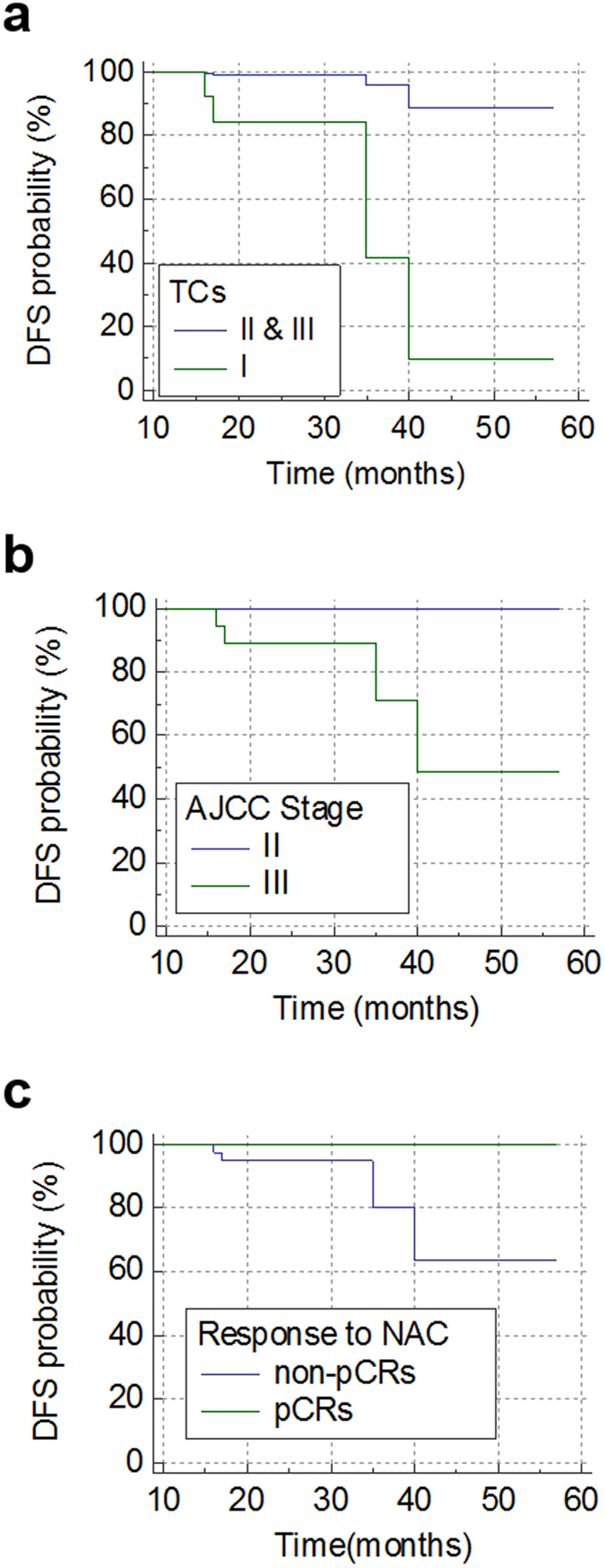
Cox regression analysis with DFS. The TC I has a hazard ratio of 19.755 (*P* = 0.010) for recurrence (**a**). Stage III had a trend of poor prognosis compared with stage II; however, this was not statistically significant (*P* > 0.05) (**b**). A pCR had a trend of a favorable prognosis compared with non-pCRs but this was not statistically significant (*P* > 0.05) (**c**). Abbreviations: DFS, disease free survival; TCs, tumor clusters; AJCC, American Joint Committee on Cancer; NAC, neoadjuvant chemotherapy; pCR, pathologic complete response.

The TC I (hazard ratio 19.755, *P* = 0.010) was identified as a prognostic factor for recurrence in univariate Cox regression analysis (Fig. 4a). Despite discriminating trends, staging (Fig. 4b) and a pCR (Fig. 4c) were not found to be significant prognostic factors in univariate Cox regression analysis because of the lack of recurrences in the stage II and pCR groups during the relatively short follow-up period (all *P* > 0.05). Further, the histopathologic parameters of ER, PgR, HER2, and Ki67 were not found to be prognostic factors in univariate Cox regression analysis (all *P* > 0.05, Supplementary Fig. S5). Multivariate Cox regression analysis with TC I and the established parameters of stage III and non-pCR showed that TC I (hazard ratio 10.246, *P* = 0.045) was an independent prognostic factor regardless of stage or achievement of a pCR.

## Discussion

We evaluated metabolic radiomics patterns in tumors and their clinical usefulness in patients with LABC. In this study, breast tumors were clustered into 3 TCs in an unsupervised manner according to their metabolic radiomics patterns. TC II, which had a moderate MTV, high SUV_max_, and high ITH, was revealed as an independent predictor of achievement of a pCR. In the survival analysis, TC I, which had a high MTV, high SUV_max_, and high ITH, was identified as an independent risk factor for recurrence when compared to the established parameters of high stage (III) and non-pCR.

A cancerous tumor is composed of a heterogeneous cell population rather than a homogeneous one, with distinct molecular and phenotypic characteristics^13^. Biological ITH is suspected to be the main reason for resistance to treatment^14^. Image-based assessment of metabolic ITH is based on the hypothesis that it may be a projection of underlying tumor biology, including glucose metabolism, necrosis, oxygenation, vascularization and angiogenesis^15^. With the heightened interest in measurement of metabolic ITH by texture analysis, a number of clinical studies have reported that TFs from PET images have more prognostic ability than conventional SUV parameters in various cancers^8–10, 16, 17^. However, investigators cannot interpret TFs in an intuitive way, because TFs merely offer a mathematical explanation of images that can be interpreted as not only heterogeneous, but also smooth, coarse, rough, or grainy^18^. Further, it has not been easy to reach a consensus regarding the parameter that best represents ITH. Therefore, an integrated radiomics approach that departs from the traditional approach is needed.

Previous PET studies in breast cancer cohorts have yielded conflicting results regarding the relationship between TFs and the histopathologic parameters of ER, PgR, and HER2^19, 20^. A recent study has reported that a TF of High-Gray-level Run Emphasis (HGRE) was significantly higher in groups of ER-negativity and PR-negativity regardless to SUV_max_^19^. However, given relatively small sample size (n = 54) and multiple comparison problem, the relationship of HGRE and hormonal receptor expression looks uncertain. Another recent study with a larger cohort (n = 171) has reported that there were not only limited relationship of TFs with hormone receptor expression, but also no additive effect of TFs discriminating breast cancer subtypes compared to SUV_max_^20^. While the previous two studies used same resampling methods of equally divided SUV ranges of tumor by 64 bins (variable bin width of SUV) and analyzed relationship of individual TFs with hormonal receptor expression, our study used a different resampling method maintaining a constant intensity resolution (fixed bin width of SUV 0.4) and integrated radiomics approach for analysis. Nonetheless, our study suggested concordant results to the latter previous study that there were no sufficient evidences that TFs are associated with hormone receptor expression. Meanwhile, our data provided concordant results to previous studies that HER2 status is not associated with TFs^19, 20^. On the other hand, Ki67, a proliferative marker, was significantly associated with unsupervised TCs in our results, which makes sense because there has been an observation of high dependency of TFs on MTV^21, 22^. In this study, our congruent results also suggest that a number of TFs were significantly correlated with MTV and/or SUV_max_, meaning that each TF should be interpreted comprehensively with consideration of MTV and/or SUV_max_.

Our data suggest that integrated metabolic radiomics has considerable potential for personalized management in LABC. For example, unsupervised TCs from metabolic radiomics can help to identify patients at higher risk for recurrence in addition to the established prognostic factors of stage and achievement of a pCR. Patients with tumors clustered as TC I might also be at high risk of recurrence. Physicians may actively consider NAC in TC II cases because of the good chance of a pCR, whereas TC III cases are less likely to achieve a pCR so are less likely to benefit NAC before surgery. In summary, use of metabolic radiomics may help in the appropriate management of individual patients and avoid the side effects of unnecessary systemic chemotherapy.

The rapid development of applications for omics data means that personalized medicine is now one step closer to becoming a reality^23^. Genomic profiling of tumors from tissue samples is being used increasingly to tailor the management strategy at the level of the individual patient. Radiomics is expected to have a role complementary to that of genomic profiling, because it has an advantage of being able to provide a non-invasive comprehensive tumor assessment that overcomes sampling error and the invasiveness of repeated biopsies^7^. Radiomics could be used as a cross-validation tool and provide information over and above that obtained from genomic profiling^24^.

SUV resampling is one of remaining issues in texture analysis, which is apparently an important methodological factor affecting the results of texture analysis. There has been two ways generally used to resample images. The most widely used method is using a fixed number of bins to divide the tumor SUV range, which results in varying intensity resolution to each case^6, 8, 25^. The other method is using a fixed bin width, which provides a constant intensity resolution to all analyzed cases^26^. In this study, we adopted a fixed bin width of SUV 0.4 in range SUV 0–25. A recent study has reported that a constant intensity resolution is more meaningful for inter- and intra-patient comparison of TFs^27^. Much validation is needed to evaluate the comparison of both two methods. Regardless, our integrated radiomics analysis method is expected to be able to use combination of both TFs obtained by the two fully different resampling methods for comparison of tumor textures on images.

There are several limitations to this study. First, the sample size for texture analysis was moderate at less than 80 cases^28^. Given that clustering is not an inferential technique, an adequate sample size for clustering is important. To avoid finding patterns in noise, we included biological validation by prediction of the likelihood of a pCR and evaluated the risk of recurrence. Not only were the results of clustering reasonably explained by several meaningful parameters (SUV_max_, MTV, and ITH-related TFs), but the biological validation suggests a clinical rationale for clustering. A multicenter trial containing much larger study cohorts is now needed to validate our results. Second, we used unsupervised clustering for this radiomics study. Supervised learning can optimize prediction of certain outcomes like histopathologic markers, response to NAC, and prognosis. Third, our results should be interpreted carefully because of the exclusion of the least metabolically active tumors. However, it should be borne in mind that delineation of tumors with little metabolic activity is usually difficult because of surrounding physiologic uptake in the breast parenchyma. In addition, we used a fixed cutoff method of SUV 2.5 can cause inaccurate tumor segmentation especially in high ITH cases. Although there was one inaccurately tumor-segmented case (Supplementary Fig. S3) with exceptional high ITH, surprisingly the case was automatically excluded during unsupervised clustering because of much different image-texture of it. In this regard, unsupervised clustering is helpful to find out extraordinary cases caused by tumor delineation error. Meanwhile, the tumor segmentation results of other cases even with high ITH were visually acceptable (Supplementary Fig. S6).

## Conclusion

LABC clustered by metabolic radiomics patterns have distinctive characteristics with regard to Ki67 expression, response to NAC, and risk of recurrence. The results of this study suggest that an integrated radiomics approach on ^18^F-FDG PET/CT has potential for personalized management for LABC.

## Methods

### Subjects

This retrospective study was approved by the Institutional Review Board at our institution. The need for written informed consent was waived. Inclusion criteria were female sex, Korean ethnicity, pretreatment ^18^F-FDG PET/CT scanning performed at the same institution before NAC for LABC from July 2009 to December 2013, and completion of NAC comprising 4 cycles of cyclophosphamide and doxorubicin or 6 cycles of adriamycin and docetaxel. One hundred cases fulfilled these criteria. Exclusion criteria were: multifocal or multicentric breast cancer (n = 7); inflammatory breast cancer (n = 4); and occult breast cancer or a tumor with so little metabolic activity that it could not be delineated with a SUV cut-off of 2.5 (n = 15). One further patient was excluded because delineation of her cancer was not possible as the primary tumor was abutting the metastatic axillary nodes too closely. Finally, 73 patients with stage LABC IIA–IIIC were enrolled for metabolic radiomics analysis. Six patients who were censored or had a recurrence before 6 months of disease-free survival (DFS) were excluded from survival analysis. One patient who did not fit into any TC was also excluded from survival analysis (Fig. 1). Immunohistochemical (IHC) parameters, including Ki67, ER, PgR, and HER2 were assessed. Ki67 >30% staining on IHC was regarded as high expression. ER or PR positivity was defined as >10% staining on IHC. HER2 positivity was defined as either strong (3+) HER2 staining on IHC or HER2 amplification identified by fluorescence *in situ* hybridization with moderate (2+) HER2 staining on IHC. The breast cancer subgroups were classified as: ER-positive or PgR-positive/HER2-negative; HER2-positive; or triple-negative. Staging was defined according to the American Joint Committee on Cancer (AJCC) system. A pCR was defined as no residual invasive cancer (AJCC ypT0/Tis ypN0). The patients were followed up for recurrence until December 2015.

### Image Acquisition and Reconstruction

Patients underwent ^18^F-FDG PET/CT on a Discovery VCT scanner (GE Medical Systems, Milwaukee, WI, USA). The blood sugar level was <120 mg/dL after at least 6 hours of fasting, and 5.18 MBq/kg of ^18^F-FDG were administered intravenously in each patient 1 hour before PET/CT scanning. CT scanning was performed at 120 kVp for attenuation correction and to obtain anatomic information. PET scans were obtained from the skull base to the upper thigh level with a 128 × 128 matrix size. The voxel size was 3.91 × 3.91 × 3.27 mm^3^. Images were reconstructed with an ordered subset expectation maximization iterative algorithm (2 iterations and 8 subsets).

### Image Texture Analysis

CGITA ver.1.4 (Chang-Gung Memorial Hospital, Taiwan) based on MATLAB v.2014a (MathWorks Inc., Natick, MA, USA) was used for analysis of the three-dimensional textures on PET images^29^. Primary tumors were delineated by a fixed SUV cut-off of 2.5^30^. Next, for calculation of TFs, the gray level was resampled by a fixed bin width method with 0.4 SUV units which was calculated from 64 grey levels of 0 to 25, to minimize the error due to variation of contrast and to improve reproducibility^6, 8, 25, 27^. Of all the methods available to compute TFs, we chose a statistics-based methodology based on the spatial distribution of gray levels^31^. Multiple matrixes were used as follows: a gray level co-occurrence matrix^32^, gray level run-length matrix^33^, gray level neighborhood intensity-difference matrix^34^, gray level size zone matrix^35^, SUV statistics, texture spectrum^36^, texture feature coding^37^, texture feature coding co-occurrence matrix^37^, and neighboring gray level dependence^38^. Finally, 109 TFs were calculated from the matrices. The matrix parameters were expressed next to the parameter name to avoid mimicking^28^. Detail on these parameters are provided in Supplementary Table S1 and in a previous report^29^. We chose NL_Entropy^GLCM^ and CV, which are generally used for measurement of ITH, to classify the extent of metabolic ITH^17, 21^. MTV (cm^3^) was defined as the volume of the tumor delineated with an SUV cut-off of 2.5. TLG (g·cm^3^/mL) was defined as the mean SUV (SUV_mean_) multiplied by the MTV of the delineated tumor. The CV was defined as the standard deviation of SUVs divided by the SUV_mean_ in a delineated tumor.

### Statistical Analysis

We used MedCalc version 14.8.1 (MedCalc Software bvba, Mariakerke, Belgium) for the statistical analysis. R version 3.2.3 (The R Foundation for Statistical Computing, Vienna, Austria) was used to construct the correlograms and heat maps. The correlations among all the 109 TFs were evaluated by Pearson correlation analysis and displayed by correlogram with hierarchical clustering. Radiomics heat maps in red to green coloring were constructed for the TF cases normalized Z-score using the Euclidean method and hierarchical clustering. Kruskal-Wallis test was used to compare representative TF values among the unsupervised TCs. The proportions of ER-positive, PgR-positive, and HER2-positive tumors, tumors with high Ki67 expression, and pCRs were compared between the unsupervised TCs using the chi-square test. The parameters included in univariate logistic regression analysis for prediction of a pCR were age, clinical AJCC stage, T stage, nodal metastasis, ER, PR, HER2, and Ki67 status, and TC. The established parameters of nodal metastasis, ER status, and HER2 status^39^, along with the parameters that were statistically significant in the univariate analysis were included in the multivariate analysis. PgR was excluded from the multivariate analysis because of its strong association with ER status. Kaplan-Meier survival analysis and the log rank test were used to compare DFS between the unsupervised TCs. A univariate Cox regression survival analysis with binary TC parameters (TC I compared with the other TC groups), including age, clinical stage, pCR, and ER, PgR, HER2, and Ki67 status, was applied to identify predictors of recurrence. Multivariate Cox regression analysis was conducted for binary TCs, disease stage, and pCRs. A *P*-value less than 0.05 (two tailed) was considered to be statistically significant. Bonferroni’s correction was applied for multiple comparison correction. Continuous values are expressed as the median and range.

## Electronic supplementary material

Supplementary table and figures
