# Supplementary material for: Metabolic Radiomics for Pretreatment 18F-FDG PET/CT to Characterize Locally Advanced Breast Cancer: Histopathologic Characteristics, Response to Neoadjuvant Chemotherapy, and Prognosis
Source: Sci Rep. 2017 May 8;7:1556. doi: 10.1038/s41598-017-01524-7 (PMC5431555; doi:10.1038/s41598-017-01524-7)
Supplement: Supplementary file 1 — Supplementary table and figures [file 41598_2017_1524_MOESM1_ESM.doc]

**Supplementary Information**

**Metabolic Radiomics for Pretreatment ^18^F-FDG PET/CT to Characterize Locally Advanced Breast Cancer: Histopathologic Characteristics, Response to Neoadjuvant Chemotherapy, and Prognosis**

Seunggyun Ha^1,2^, Sohyun Park^1,3^, Ji-In Bang^1,3^, Eun-Kyu Kim^4^, Ho-Young Lee^1,3,5^*

^1^Department of Nuclear Medicine, Seoul National University College of Medicine, Seoul, Korea

^2^Department of Molecular Medicine and Biopharmaceutical Sciences, Graduate School of Convergence Science and Technology, and College of Medicine or College of Pharmacy, Seoul National University

^3^Department of Nuclear Medicine, Seoul National University Bundang Hospital, Gyeonggi-do, Korea

^4^Department of General Surgery, Seoul National University Bundang Hospital, Gyeonggi-do, Korea

^5^Cancer Research Institute, Seoul National University, Seoul, Korea

**Corresponding author:** Ho-Young Lee, MD, PhD

Department of Nuclear Medicine, Seoul National University Bundang Hospital, 82 Gumi-ro 173 Beon-gil Bungdang-gu, Gyeonggi-do 463-707, South Korea

Tel: +82-31-787-7679

Fax: +82-31-787-4018

E-mail: [debobkr@gmail.com](mailto:debobkr@gmail.com)

**First author:** Seuggyun Ha, MD

^1^Department of Nuclear Medicine, Seoul National University College of Medicine, 101 Daehak-ro, Jongno-gu, 03080, Seoul, Korea

Tel: +82-2-2072-3341

Fax: +82-2-745-7690

E-mail: seunggyun.ha@gmail.com

**Supplemental Table S1.** Texture Features

| Matrix | Texture Feature Name | Abbreviations | Orders in correlogram | Orders in heatmap |
| --- | --- | --- | --- | --- |
| GLCM | Second Angular Moment | SAM^GLCM^ | c22 | h95 |
|  | Contrast | Contrast^GLCM^ | c57 | h24 |
|  | Entropy | Entropy^GLCM^ | c8 | h6 |
|  | Homogeneity | Homogeneity^GLCM^ | c34 | h105 |
|  | Dissimilarity | Dissimilarity^GLCM^ | c93 | h56 |
|  | Inverse difference moment | IDM^GLCM^ | c35 | h104 |
| GLRM | Short run emphasis | SRE^GLRM^ | c13 | h1 |
|  | Long run emphasis | LRE^GLRM^ | c40 | h86 |
|  | Intensity variability | IV^GLRM^ | c42 | h87 |
|  | Run-length variability | RLV^GLRM^ | c33 | h103 |
|  | Run percentage | RP^GLRM^ | c108 | h49 |
|  | Low-intensity run emphasis | LIRE^GLRM^ | c109 | h50 |
|  | High-intensity run emphasis | HIRE^GLRM^ | c53 | h15 |
|  | Low-intensity short-run emphasis | LISRE^GLRM^ | c107 | h48 |
|  | High-intensity short-run emphasis | HISRE^GLRM^ | c54 | h16 |
|  | Low-intensity long-run emphasis | LILRE^GLRM^ | c41 | h85 |
|  | High-intensity long-run emphasis | HILRE^GLRM^ | c52 | h17 |
| GLNIDM | Coarseness | Coarseness^GLNIDM^ | c5 | h13 |
|  | Contrast | Contrast^GLNIDM^ | c12 | h3 |
|  | Busyness | Busyness^GLNIDM^ | c15 | h78 |
|  | Complexity | Complexity^GLNIDM^ | c55 | h19 |
|  | Strength | Strength^GLNIDM^ | c56 | h18 |
| GLSZM | Short-zone emphasis | SZE^GLSZM^ | c72 | h35 |
|  | Large-zone emphasis | LZE^GLSZM^ | c46 | h92 |
|  | Intensity variability | IV^GLSZM^ | c23 | h96 |
|  | Size-zone variability | SZV^GLSZM^ | c94 | h57 |
|  | Zone percentage | ZP^GLSZM^ | c14 | h2 |
|  | Low-intensity zone emphasis | LIZE^GLSZM^ | c4 | h12 |
|  | High-intensity zone emphasis | HIZE^GLSZM^ | c60 | h21 |
|  | Low-intensity short-zone emphasis | LISZE^GLSZM^ | c10 | h5 |
|  | High-intensity short-zone emphasis | HISZE^GLSZM^ | c61 | h22 |
|  | Low-intensity large-zone emphasis | LILZE^GLSZM^ | c47 | h93 |
|  | High-intensity large-zone emphasis | HILZE^GLSZM^ | c45 | h91 |
| GLCM | Normalized Second Angular Moment | NL_SAM^GLCM^ | c1 | h9 |
|  | Normalized Contrast | NL_Contrast^GLCM^ | c59 | h23 |
|  | Normalized Entropy | NL_Entropy^GLCM^ | c105 | h71 |
|  | Normalized Homogeneity | NL_Homogeneity^GLCM^ | c2 | h10 |
|  | Normalized Dissimilarity | NL_Dissimilarity^GLCM^ | c66 | h26 |
|  | Normalized Inverse difference moment | NL_IDM^GLCM^ | c3 | h11 |
|  | Correlation | Correlation^GLCM^ | c103 | h69 |
| SUV statistics | Minimum SUV | SUV_min_ | c7 | h8 |
|  | Maximum SUV | SUV_max_ | c64 | h29 |
|  | Mean SUV | SUV_mean_ | c67 | h27 |
|  | SUV Variance | Variance | c58 | h20 |
|  | SUV SD | SD | c63 | h30 |
|  | Coefficient of variance | CV | c104 | h70 |
|  | SUV Skewness | Skewness | c50 | h79 |
|  | SUV Kurtosis | Kurtosis | c44 | h94 |
|  | SUV bias-corrected Skewness | Skewness_bias-corrected_ | c51 | h80 |
|  | SUV bias-corrected Kurtosis | Kurtosis_bias-corrected_ | c49 | h81 |
|  | Total legion glycolysis | TLG | c91 | h61 |
|  | Metabolic tumor volume | MTV | c24 | h98 |
|  | Entropy | Entropy | c106 | h72 |
|  | SULpeak | SULpeak | c65 | h28 |
|  | Surface area | Surface area | c26 | h99 |
|  | Asphericity | Asphericity | c36 | h83 |
|  | Asphericity 2 | Asphericity 2 | c37 | h84 |
|  | Asphericity 3 | Asphericity 3 | c6 | h14 |
|  | Surface mean SUV 1 | Surface SUV_mean_ 1 | c74 | h37 |
|  | Surface total SUV 1 | Surface total 1 | Ch28 | h101 |
|  | Surface SUV entropy 1 | Surface entropy 1 | c77 | h40 |
|  | Surface SUV variance 1 | Surface variance 1 | c69 | h33 |
|  | Surface SUV SD 1 | Surface SD 1 | c70 | h31 |
|  | Surface SUV NSR 1 | Surface NSR 1 | c71 | h32 |
|  | Surface mean SUV 2 | Surface SUV_mean_ 2 | c29 | h100 |
|  | Surface total SUV 2 | Surface total 2 | c30 | h107 |
|  | Surface SUV entropy 2 | Surface entropy 2 | c78 | h41 |
|  | Surface SUV variance 2 | Surface variance 2 | c85 | h52 |
|  | Surface SUV SD 2 | Surface SD 2 | c87 | h58 |
|  | Surface SUV NSR 2 | Surface NSR 2 | c88 | h59 |
|  | Surface mean SUV 3 | Surface SUV_mean_ 3 | c38 | h89 |
|  | Surface total SUV 3 | Surface total 3 | c43 | h88 |
|  | Surface SUV entropy 3 | Surface entropy 3 | c76 | h39 |
|  | Surface SUV variance 3 | Surface variance 3 | c68 | h34 |
|  | Surface SUV SD 3 | Surface SD 3 | c98 | h67 |
|  | Surface SUV NSR 3 | Surface NSR 3 | c99 | h68 |
|  | Surface mean SUV 4 | Surface SUV_mean_ 4 | c25 | h97 |
|  | Surface total SUV 4 | Surface total 4 | c31 | h108 |
|  | Surface SUV entropy 4 | Surface entropy 4 | c75 | h38 |
|  | Surface SUV variance 4 | Surface variance 4 | c86 | h51 |
|  | Surface SUV SD 4 | Surface SD 4 | c89 | h62 |
|  | Surface SUV NSR 4 | Surface NSR 4 | c90 | h63 |
|  | SUVmean_prod_asphericity | SUVmean_prod_A | c100 | h66 |
|  | SUVmax_prod_asphericity | SUVmax_prod_A | c101 | h65 |
|  | Entropy_prod_asphericity | Entropy_prod_A | c39 | h90 |
|  | SULpeak_prod_asphericity | SULpeak_prod_A | c102 | h64 |
|  | SUVmean_prod_surface_area | SUVmean_prod_SA | c95 | h55 |
|  | SUVmax_prod_surface_area | SUVmax_prod_SA | c96 | h53 |
|  | Entropy_prod_surface_area | Entropy_prod_SA | c27 | h102 |
|  | SULpeak_prod_surface_area | SULpeak_prod_SA | c97 | h54 |
| TS | Max spectrum | Spectrum_max_^TS^ | c79 | h42 |
|  | Black-white symmetry | BW_symmetry^TS^ | c21 | h109 |
| TFC | Coarseness | Coarneness^TFC^ | c11 | h4 |
|  | Homogeneity | Homogeneity^TFC^ | c48 | h82 |
|  | Mean convergence | MC^TFC^ | c80 | h44 |
|  | Variance | Variance^TFC^ | c19 | h74 |
| TFCCM | Second angular moment | SAM^TFCCM^ | c82 | h47 |
|  | Contrast | Contrast^TFCCM^ | c20 | h73 |
|  | Entropy | Entropy^TFCCM^ | c16 | h76 |
|  | Homogeneity | Homogeneity^TFCCM^ | c83 | h46 |
|  | Intensity | Intensity^TFCCM^ | c81 | h43 |
|  | Inverse difference moment | IDM^TFCCM^ | c84 | h45 |
|  | Code Entropy | CE^TFCCM^ | c17 | h77 |
|  | Code Similarity | CS^TFCCM^ | c62 | h25 |
| NGLD | Small number emphasis | SNE^NGLD^ | c73 | h36 |
|  | Large number emphasis | LNE^NGLD^ | c18 | h75 |
|  | Number nonuniformity | NNU^NGLD^ | c92 | h60 |
|  | Second moment | SM^NGLD^ | c32 | h106 |
|  | Entropy | Entropy^NGLD^ | c9 | h7 |
| Abbreviations: GLCM, gray level co-occurrence matrix; GLRM, gray level run-length matrix; GLNIDM, gray level neighborhood intensity-difference matrix; GLSZM, gray level size zone matrix; SUV, standardized uptake value; TS, texture spectrum; TFC, texture feature coding; TFCCM, texture feature coding co-occurrence matrix; NGLD, neighboring gray level dependence; NL, normalized | | | | |

**
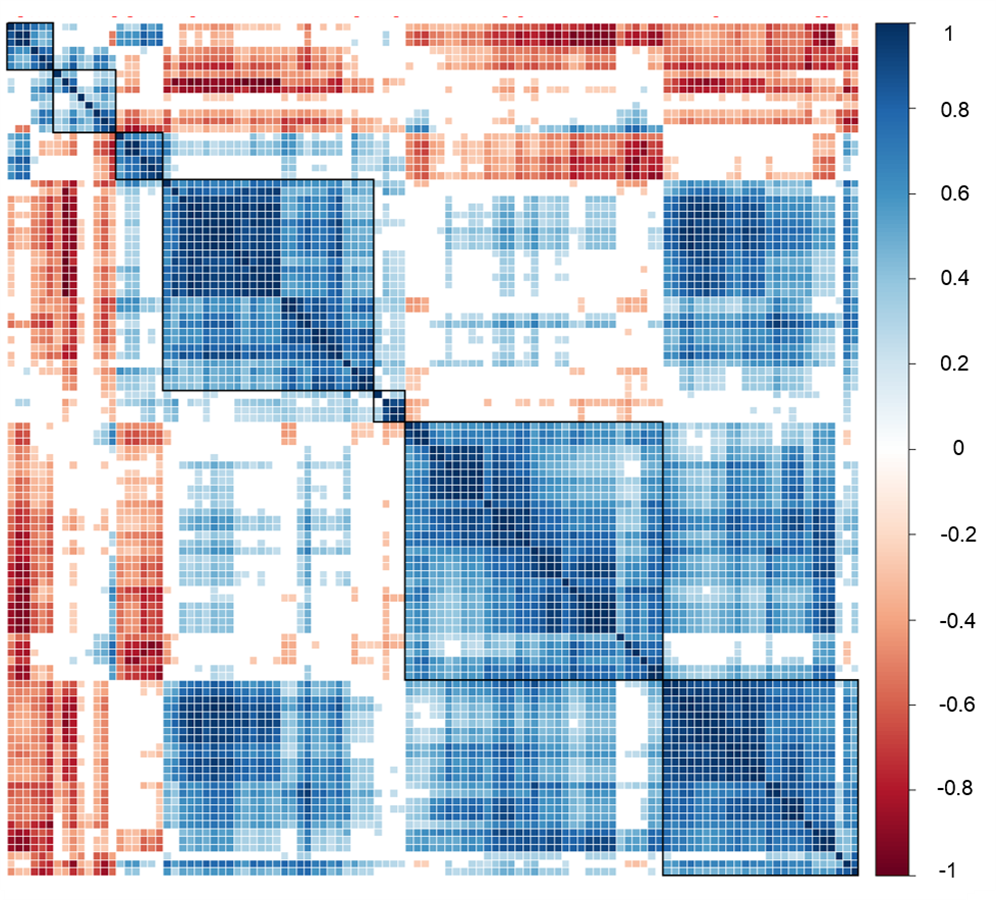
**

**Supplemental Figure S1. Correlogram of texture features (TFs) before multiple comparison correction (*P* < 0.05).** Almost TFs were correlated to one another. Eighty-eight TFs (80.7%) were correlated to SUV_max_, and 61 TFs (56.0%) were correlated to metabolic tumor volume.


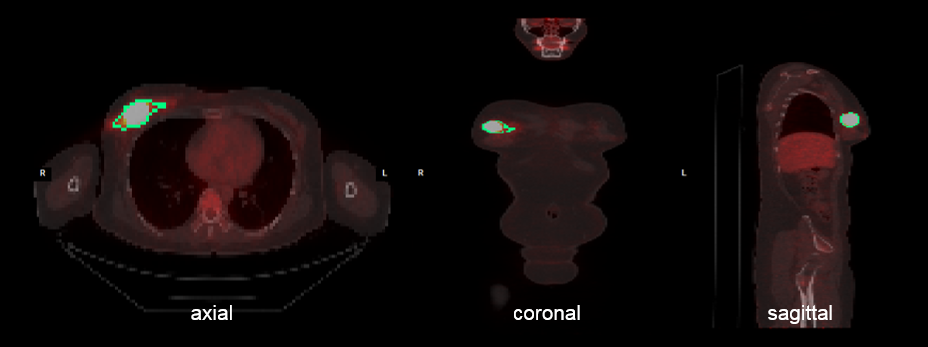


**Supplemental Figure S2. Exceptional case not included in any clusters.** The transverse, coronal, and sagittal images (color range: SUV 0-10) showed the patient (F/30) not included in any clusters due to inaccurate tumor delineation. The tumor was classified as triple-negative breast cancer and high Ki67 expression of 95%. PET parameters were SUV_max_ of 49.66, metabolic tumor volume (MTV) of 44.26, total lesion glycolysis (TLG) of 675.89, coefficient of variance (CV) of 0.91, and normalized entropy on gray level co-occurrence matrix (NL_Entropy^GLCM^) of 7.39. The tumor achieved a pCR after neoadjuvant chemotherapy. No recurrence was occurred during follow-up.

**
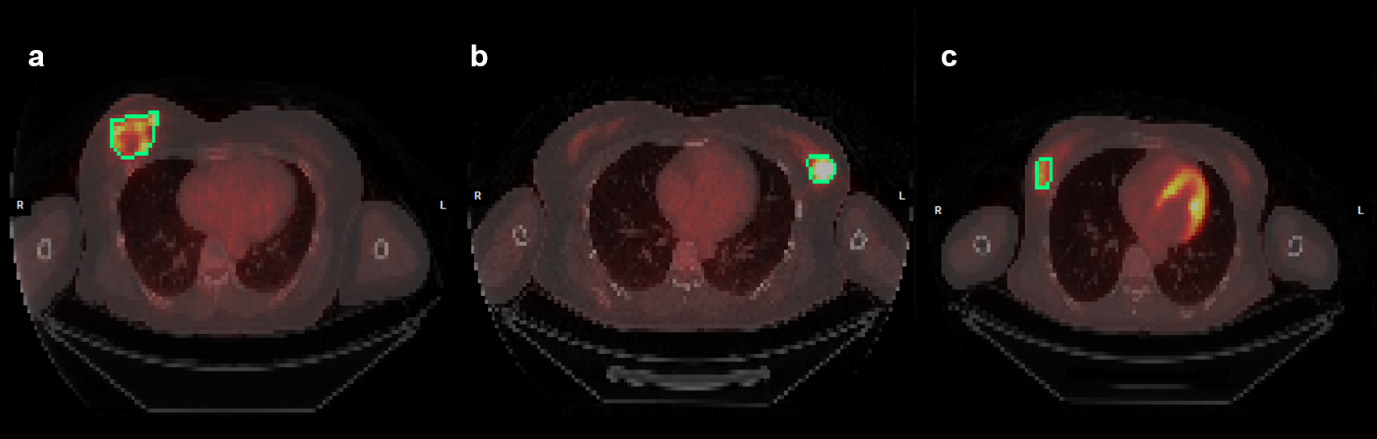
**

**Supplemental Figure S3. Representative cases of the unsupervised tumor clusters (TCs).** Triple negative infiltrative ductal carcinoma (IDC) of 44 year-old woman was clustered in the TC I. Clinical staging was T3 and N2. The PET/CT scan showed SUV_max_ of 13.05, MTV of 99.74, TLG of 499.47, CV of 0.45, and NL_Entropy^GLCM^ of 5.77. The tumor did not achieve a pCR after NAC. Tumor recurrence was observed 40 months after surgical tumor removal (a). Triple negative IDC of 38 year-old woman was clustered in the TC II. Clinical staging was T2 and N0. The PET/CT scan showed SUV_max_ of 16.98, MTV of 15.67, TLG of 109.42, CV of 0.56, and NL_Entropy^GLCM^ of 6.18. The tumor achieved a pCR after NAC. Tumor recurrence was not observed till 31 months from operation (b). Triple negative IDC of 38 year-old woman was clustered in the TC III. Clinical staging was T2 and N0. The PET/CT scan showed SUV_max_ of 6.86, MTV of 9.33, TLG of 38.26, CV of 0.27, and NL_Entropy^GLCM^ of 4.35. The tumor failed to achieve a pCR. Tumor recurrence was not observed till 21 months from operation (c).

**
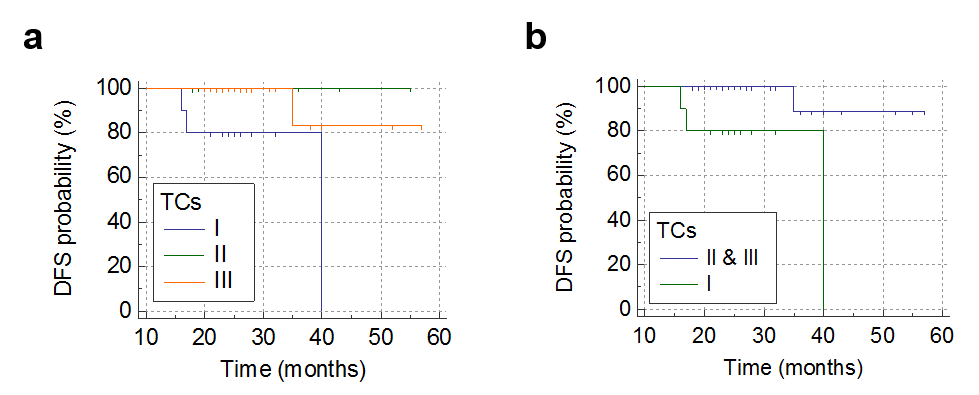
**

**Supplemental Figure S4.** **Kaplan-Meier survival graphs of tumor clusters (TCs).** Graphs show poorer prognosis of the TC I than other TCs. The unsupervised 3 TCs had significantly different DFS (*P* = 0.001) (a). The results of Kaplan-Meier survival analysis for binary TCs as the TC I versus others was also significant (*P* < 0.001) (b).

**
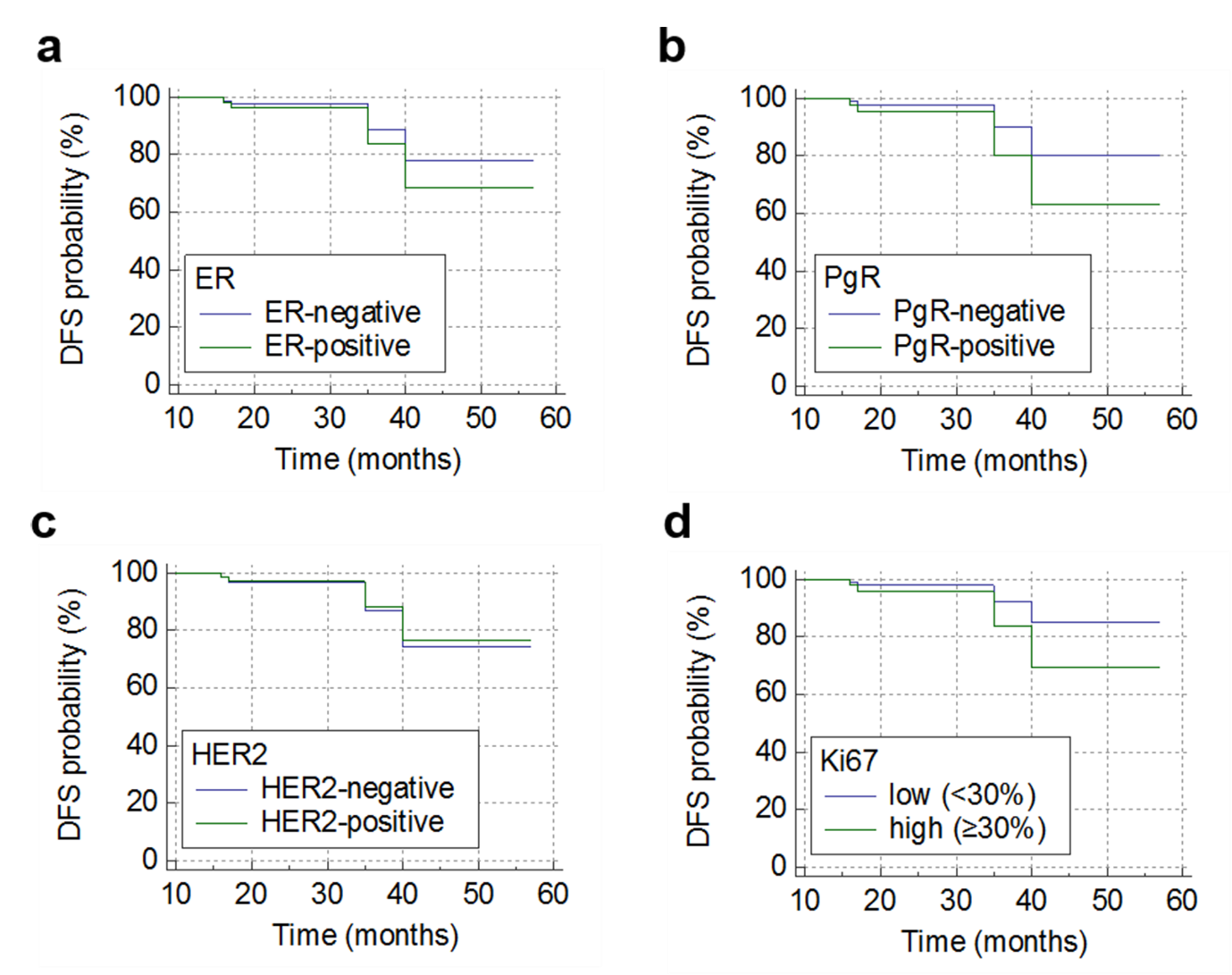
**

**Supplemental Figure S5. Cox-regression analysis of histopathologic markers.** ER (a), PgR (b), HER2 (c), and Ki67 (d) status were not prognostic factors for recurrence in Cox-regression analysis.

**
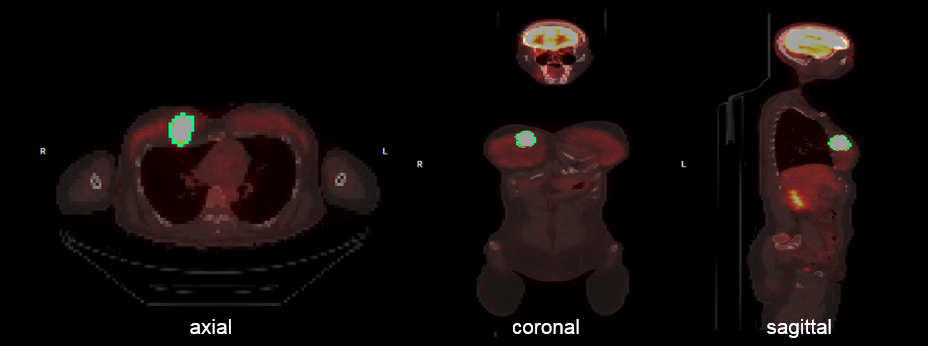
Supplemental Figure S6. Appropriate tumor delineation in the case of high intratumoral heterogeneity.** The images showed an example of high ITH (2^nd^ highest level of NL_EntropyGLCM of 6.97 & 4^th^ highest level of CV of 0.6034) but well delineated tumor.
